# Diffusion Rates and Dispersal Patterns of Unfed *versus* Recently Fed Bed Bugs (*Cimex lectularius* L.)

**DOI:** 10.3390/insects6040792

**Published:** 2015-09-24

**Authors:** Jerome Goddard, Michael Caprio, Jerome Goddard

**Affiliations:** 1 Department of Biochemistry, Molecular Biology, Entomology, and Plant Pathology, Mississippi State University, Mississippi State, MS 39762, USA; E-Mail: mac24@msstate.edu; 2 Department of Mathematics and Science, Auburn University Montgomery, Montgomery, AL 36124, USA; E-Mail: jgoddard@aum.edu

**Keywords:** bed bugs, locomotor activity, host-seeking, aggregation activity

## Abstract

Bed bug problems have been increasing since the 1980s, and accordingly, there have been intensive efforts to better understand their biology and behavior for control purposes. Understanding bed bug diffusion rates and dispersal patterns from one site to another (or lack thereof) is a key component in prevention and control campaigns. This study analyzed diffusion rates and dispersal patterns in a population of bed bugs, recently fed and unfed, in both one-dimensional and two-dimensional settings. When placed in the middle of a 71 cm × 2.7 cm artificial lane, approximately half of the bugs regardless of feeding status stayed at or near the release point during the 10 min observation periods, while about a fourth of them walked to the end of the lane. When placed in the middle of an arena measuring 51 cm × 76 cm and allowed to walk in any direction, approximately one-fourth of bed bugs, fed or unfed, still remained near their release point (no significant difference between fed or unfed). As for long-distance dispersal, 11/50 (22%) of recently fed bed bugs moved as far as possible in the arena during the 10 min replications, while only 2/50 (4%) unfed bed bugs moved to the maximum distance. This difference was significantly different (*p* < 0.0038), and indicates that unfed bed bugs did not move as far as recently fed ones. A mathematical diffusion model was used to quantify bed bug movements and an estimated diffusion rate range of 0.00006 cm^2^/s to 0.416 cm^2^/s was determined, which is almost no movement to a predicted root mean squared distance of approximately 19 cm per 10 min. The results of this study suggest that bed bugs, upon initial introduction into a new area, would have a difficult time traversing long distances when left alone to randomly disperse.

## 1. Introduction

Bed bugs are blood-feeding pests of various warm-blooded animals such as humans, bats, birds, and pets [1,2,3,4]. These parasites have been resurging in the U.S. since the 1980’s [5,6], being increasingly reported inside U.S. hotel rooms, dorms, and apartments [7,8,9]. Health effects from bed bug bites include pruritic lesions and rashes, bullae, and rarely systemic allergic reactions [6,10,11,12,13,14,15]. Other than some concern over possible transmission of Chagas’ disease [16], there is currently little evidence supporting significant disease transmission by bed bugs [17].

Control of bed bugs is a multi-million dollar industry, and unfortunately, much of the basic science about bed bug behavior underpinning pest control efforts is lacking. Questions about bed bug dispersal (if so, and to what extent) remain incompletely answered, and mathematical analyses of diffusion rates of bed bugs have not been reported. Much of bed bug movement concerns host-seeking or harborage-seeking. Several studies have examined bed bug locomotor activity in regard to these two aspects, as well as the influence of host signals and cues on movement [18,19,20]. In many such studies, either video or time-lapse photography was used to track movement of bed bugs inside an arena [19,21,22]. Of the many approaches reported in the literature to study animal dispersal, the diffusion framework has been of enormous value in both empirical and theoretical investigations of spatial aspects [23]. A detailed history of the diffusion framework is available [24,25,26,27,28,29,30,31]. The coefficients of a diffusion model express quantities measurable by direct observation of individual movement behavior. In fact, the diffusion coefficient of the model and its dependence on factors such as environment or conspecific density provide a concise and standardized measure of the dispersal rate/pattern of the organisms which can be used in several other models [23,32]. Specifically related to studying bed bug spatio-temporal distribution, the diffusion framework intrinsically provides for analyses yielding insight into the “patch-level” consequences of individual-based mechanistic behavior. In other words, the patch level consequence of individual bed bug behavior assumptions can be validated empirically, as well as investigated theoretically via reaction diffusion models which incorporate density-dependent effects such as births and deaths, all within the diffusion framework. The aim of this project was to study the diffusion rates and dispersal patterns in a population of bed bugs, some recently fed and some unfed, in both one-dimensional and two-dimensional arenas.

## 2. Methods

### 2.1. Bed Bugs, Arena, and Substrates

**Figure 1:**
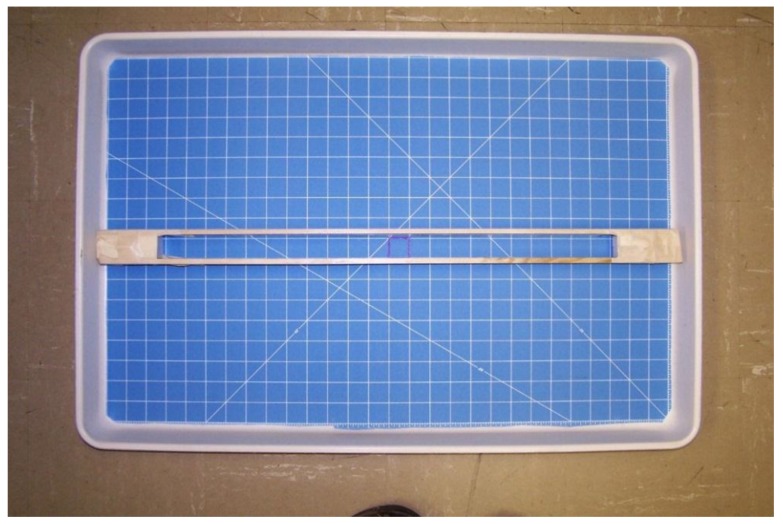
One-dimensional arena set-up. Bed bugs were released inside the marked square.

**Figure 2:**
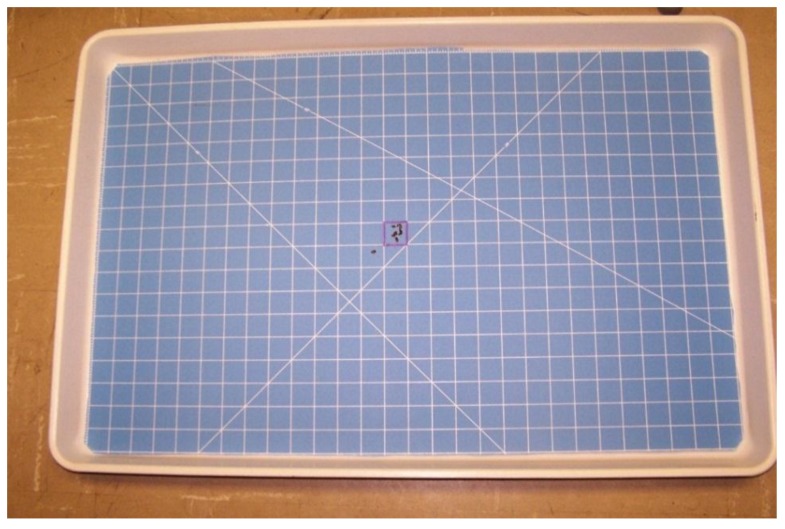
Two-dimensional arena set-up showing bed bug positions almost immediately after release.

This work was performed in the Department of Biochemistry, Molecular Biology, Entomology, and Plant Pathology, Mississippi State University, between 18 April 2014 and 5 June 2014. Bed bugs used in this study were the Ft. Dix, Harold Harlan strain. Bed bugs were stored, and all experiments conducted in, a laboratory with approximately 22 °C, 50% relative humidity, and a photoperiod of 8:16 (L:D). There were no windows in the lab, and thus no exposure to sunlight. All bed bug dispersal observations were made during daytime, inside the lab, with the lights turned on. The arena consisted of a Tailor^®^ rotary cutting mat (Hobby Lobby Stores, Inc., Oklahoma City, OK, USA) 61 cm × 91 cm trimmed to fit inside a 51 cm × 76 cm molded fiber glass tray (Fisher Scientific, Hampton, NH, USA) (Figure 1). To approximate a one-dimensional path, two yardsticks (Lowe’s Stores, Inc., Winston-Salem, NC, USA) were cut to 71 cm each and taped together with a spacer at both ends and with 2.7 cm between them, corresponding roughly to the width of the squares engraved on the rotary cutting board substrate (Figure 1). Note: since the path was wider than the width of an adult bed bug, this was not a true one-dimensional trough. However, invariably, upon release bed bugs went to the sides and walked straight forward and backward. For the two-dimensional experiment, the entire substrate was available for bed bugs to crawl in any direction (Figure 2).

### 2.2. Protocol

Bed bugs in two “fed” states were utilized in this study: recently fed (1 week since feeding), and unfed (7 weeks since feeding). For each experiment, 10 (equal number of male and female) adult bed bugs were placed in a four-dram plastic vial which was inverted over the center square of the substrate. After one minute, the vial was removed, allowing the bugs to crawl about freely. The authors then left the room to minimize host cues such as heat, CO_2_, shadows, *etc.*, and observed bed bug dispersal in the arena from outside the room in the hallway. Bed bug dispersal was video recorded for 10 min, after which the location and sex of each bug was recorded. All bed bugs were removed and the substrate wiped down with 95% ethanol. This procedure was repeated 5 times for each condition—one-dimensional and two-dimensional, with both recently fed and unfed bed bugs—for a total of 20 replicates. During each replication, if any bugs reached the edges (boundary) of the arenas, the authors quickly re-entered the room and retrieved those bugs (all such bugs were considered “emigrated” or “lost” from that point on in the replication).

### 2.3. Calculation of Diffusion Rates

A detailed discussion of diffusion models has been previously published [31] and we closely followed the principles outlined therein. In this study, density-distance data were obtained by examining 30 still frames, k∈{1,2,…,30}, from each 10-min video (20 total videos) at 20 s increments. Based on the grid size of the arenas, this data was recorded in 2.7 cm line segments in the one-dimensional arena and in 2.7 cm × 2.7 cm squares in the two-dimensional arena. We used a diffusion model to quantify bed bug movements from this density-distance data. Observation of the individual tracks of bed bugs indicated that they made many short movements in random directions which suggested the validity of the use of diffusion models to quantify the movement of bed bugs in this study. For the one-dimensional arena, we used a one-dimensional linear domain, $\Omega_{1}$, measuring approximately from −22.86 cm to 22.86 cm with the release point as the origin. For the two-dimensional arena, we used a two-dimensional rectangular domain, $\Omega_{2}$ with measuring approximately from −35.56 cm to 33.02 cm in width and −20.32 cm to 22.86 cm in length with the release point as the origin. In order to match the assay as closely as possible, we chose the standard diffusion model with u(t,x) measuring population density at time t and location x or (x,y):

(1) $$\{\begin{array}{c} u_{t}=Du_{xx};x\in \Omega_{1}\\ u=0;x\in \partial \Omega_{1}\; \end{array}\;$$

(2) $$\{\begin{array}{c} u_{t}=D\left(u_{xx}+u_{yy}\right);\left(x,y\right)\in \Omega_{2}\\ u=0;\left(x,y\right)\in \partial \Omega_{2} \end{array}$$

for the one- and two-dimensional domains, respectively. The boundary conditions listed in Equations (1) and (2) are called absorbing (or Dirichlet in the mathematical literature) and are appropriate since bed bugs were immediately removed from the arena upon reaching the boundary. Here, D>0 is the diffusion rate measured in cm^2^/s, regardless of dimension of the domain. Two different initial conditions were employed in the diffusion models.

The first initial condition (IC_1_) represented an area release at time t=0 (k=1) with (1) a uniform density of 10 in the interval [−1.35, 1.35] centered at the origin and a density of 0 elsewhere for $\Omega_{1}$ and (2) a uniform density of 10 in a 2.7 cm square centered at the origin and a density of 0 elsewhere for $\Omega_{2}$. This initial condition is used for samples k∈{2,3,…,30} and assumes that bed bugs start from time t=0 and then disperse to the time t=20(k−1). The corresponding diffusion rate that provides the best fit of the numerical solution of the model to the density-distance data is then a measure of the bed bug’s dispersal during the 20(k−1) seconds interval. The use of (IC_1_) has been recommended by Turchin and others when animals are instantaneously released at a uniform density in an area as opposed to the usual instantaneous point release [31].

The second initial condition (IC_2_) was chosen in order to estimate the diffusion rate over each 20 s interval in the 10 min study window. In this case, when ascertaining the diffusion rate for a sample, k∈{2,3,…,30}, (IC_2_) was chosen to be the actual density distribution recorded from the previous sample k−1. The diffusion rate yielding the best fit of the solution of the diffusion model to the density data then provides a measure of the dispersal of the bed bugs during the 20 s period from 20(k−2) to 20(k−1) in seconds.

Due to the nature of the initial conditions and the absorbing boundary condition, the diffusion models were solved numerically. In particular, a standard finite difference scheme was implemented in Mathematica 10.1 (Wolfram Inc., Champaign, IL, USA, 2015) to numerically solve both initial-boundary value problems. The numerical solutions of (1) and (2) with both initial conditions (IC_1_) and (IC_2_) were then fit to density-distance data using a nonlinear least squares algorithm implemented in Mathmatica 10.1 to provide an estimate of the diffusion rates for each sample across all reps [31]. The nonlinear least squares algorithm minimized the sum of squares (SS) differences between the density-distance data and the numerical solution by:

(1) selecting a search range for estimates of the diffusion rate, $D_{\text{min}}$ and $D_{\text{max}}$ (for the initial range for all reps, $D_{\text{min}}=0.00001$ to $D_{\text{max}}=0.5$ was chosen based on *a priori* observations that indicated this was the optimal range for possible diffusion rates)
(2) creating a mesh of the interval $\left(D_{min},\;D_{max}\right)$ with 100 estimates of the diffusion rate, *i.e.*, $D_{i}=D_{\text{min}}+i*D_{step};\;D_{step}=\frac{D_{max}-D_{min}}{100},\;i=\left\{1,2,\ldots ,100\right\}$, (with the initial case giving $D_{step}=0.005$ for $D_{\text{min}}=0.00001$ and $D_{\text{max}}=0.5$);
(3) finding the smallest SS difference, say k, and refining the search range to $D_{\text{min}}=D_{k-1}$ and $D_{\text{max}}=D_{k+1}$;
(4) repeating steps (2)–(3) until $D_{\text{max}}-D_{min}\leq 10^{-7}$.

The diffusion rates estimated from the model using (IC_1_) were then averaged over all samples in a rep to arrive at an estimate of the diffusion rate of the bed bugs for that rep. The diffusion rates estimated from the model using (IC_2_) are designed to give an estimate of the diffusion rate of the bed bugs over each of the 20 s sample intervals and are thus not averaged across time.

### 2.4. Statistical Analyses

Distance traveled by bed bugs (means of fed *vs.* unfed bed bugs), emigration (reached the edge of the arenas) data, and mean diffusion rates calculated using (IC_1_) were analyzed using a two-tailed *t*-test. A repeated measures ANOVA in the R statistical program (The R Foundation, Vienna, Austria, 2009) was used for analysis of the time-dependent diffusion rates obtained using the initial condition (IC_2_) with time as the “repeated (within) factor” and feeding status as the “between factor”. For more in-depth analysis of distance traveled *versus* feeding status, the sm.density.compare function from the sm package [33] in the R statistical program (R Statistical Package 2009) was used to compare the distribution of distance between the two levels of feeding status. The sm package uses kernel density estimates that are similar to histograms but have several improvements—they are smoothed (continuous) and have no endpoints [33,34]. Both histograms and kernel density estimates are sensitive to the bandwidth used. The default optimal value for bandwidth calculated by the sm.density function was used for these comparisons. The sm.density.compare function compares for equality between two distributions using a boostrap analysis (5000 bootstraps were used).

## 3. Results and Discussion

**Figure 3:**
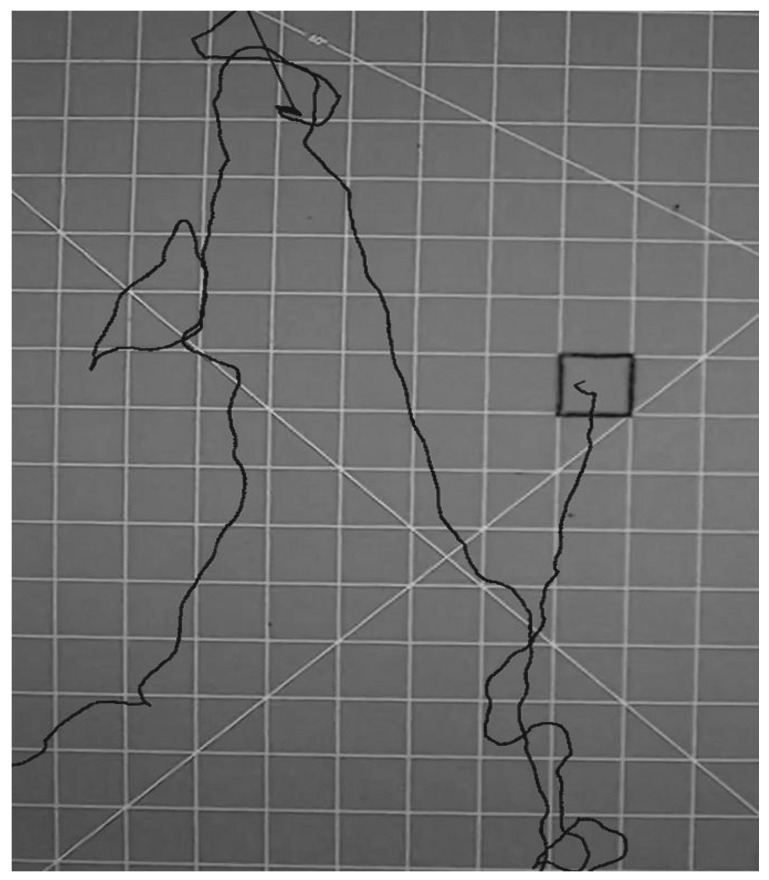
Typical bed bug searching pattern observed within the two-dimensional arena as plotted using time-lapse photography (this was the track of an unfed bug).

Bed bugs disperse from one place to another for a variety of reasons such as searching for food, mates, or harborage. In these experiments which were conducted in a lighted arena in the absence of any known host cues, we assumed their locomotor activity was primarily due to harborage or host seeking. We acknowledge that their behavior might be due to a “panic” or “flight” dispersal resulting from being released into an open, lighted arena (however, the dispersal patterns we saw closely resembled those previously reported for normal host-seeking, Figure 3). Dispersal patterns observed in this experiment generally consisted of relatively straight lines for approximately 10–25 cm, followed by “looping back,” to go back where they came from or go in a different direction (Figure 3). These observed movement patterns were similar to those seen by Suchy and Lewis who described them as a “tortuous meander” [22].

### 3.1. One-Dimensional Dispersal

**Figure 4:**
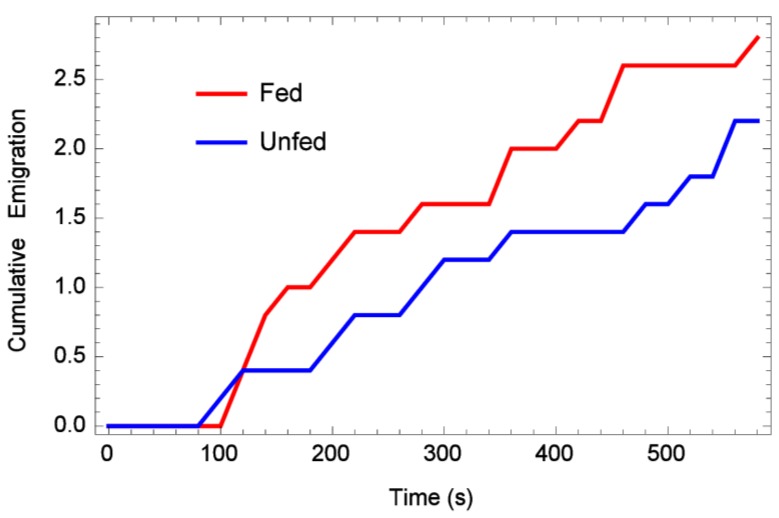
Mean cumulative emigration rates over time (bugs that reached the edge), one-dimensional arena.

When placed in the middle of an artificial lane, approximately half of the bugs regardless of feeding status stayed at or near the release point during the 10 min observation periods (Table 1), while about a fourth of them walked to the end of the lane. There was no significant difference between the numbers of fed or unfed bed bugs which moved (or did not move) in this experiment (sm.density.compare bootstrap, *n* = 5000, *p* = 0.771). The “looping back” or “tortuous meander” dispersal pattern was restricted in these experiments due to the narrow lanes, therefore bed bugs pursued a straight path much further. When cumulative emigration (number bed bugs lost by reaching the edge of the arena) per 20 s time period was analyzed, it appeared that fed bugs reached the edge of the arenas more often than unfed ones (Figure 4). However, a *t*-test on the total number of emigrants per rep, fed *vs.* unfed, yielded *p* < 0.647. This lack of statistical significance might be related to the fact that the one-dimensional arena was so much narrower.

**Table 1:** Dispersal of bed bugs in an arena over a 10-min period (all reps combined).

**Number that Moved Very Little (<4 cm)**		
ARENA TYPE	FED	UNFED
one-dimensional	9♀ 16♂ (50%)	14♀ 13♂ (54%)
two-dimensional	5♀ 8♂ (26%)	4♀ 8♂ (24%)
**Number that moved as far as possible (22.5–25 cm one-dimensional or 34–41 cm two dimensional)** *		
ARENA TYPE	FED	UNFED
one-dimensional	10♀ 5♂ (30%)	7♀ 5♂ (24%)
two-dimensional	6♀ 5♂ (22%)	2♀ (4%)

* Depended on direction the bugs crawled. Straight to the edge vertically was 34 cm; diagonally to the edge (longest distance possible) was 41 cm.

### 3.2. Two-Dimensional Dispersal

**Figure 5:**
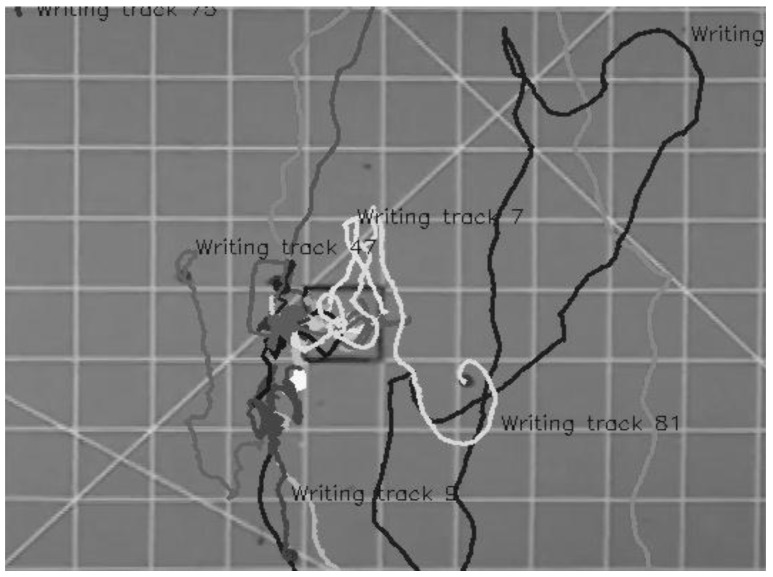
Bed bug movements as measured by time-lapse photography. Approximately 25% of bed bugs in the two-dimensional arena (fed or unfed) did not move more than 4 cm from the release point (For example, the white track).

**Figure 6:**
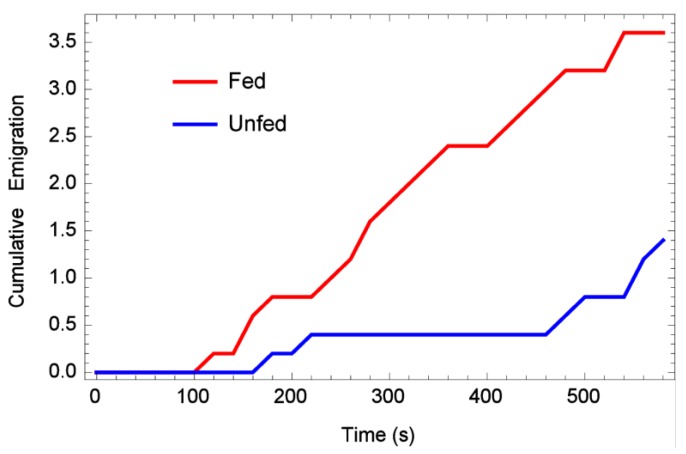
Mean cumulative emigration rates over time (bugs that reached the edge), two-dimensional arena.

When placed in the middle of an arena and allowed to walk in any direction, approximately one-fourth of bed bugs, fed or unfed, still remained near their release point (no significant difference between fed or unfed) (Figure 5). This compares with another study which showed 84% of bed bugs never moved from their release point in an arena during the observation period [22]. As for long-distance dispersal, 11/50 (22%) of recently fed bed bugs moved as far as possible in the arena during the 10 min replications, while only 2/50 (4%) unfed bed bugs moved to the maximum distance. This difference was significantly different (*p* < 0.0038), and indicates that unfed bed bugs did not move as far as recently fed ones. This can also be seen when cumulative emigration (number bed bugs lost by reaching the edge of the arena) per 20 s time period was analyzed (Figure 6). In addition, a *t*-test on total number of emigrants per rep, fed and unfed, yielded *p* < 0.01, indicating that fed bed bugs left the arena more frequently than unfed ones.

An in-depth analysis of distance moved *versus* feeding status using the sm.density.compare package of the R statistical environment also showed that unfed bed bugs moved less (*p* < 0.041) (Table 1). These data are consistent with a previous study [20] in which bed bugs within one-week of feeding were more active than those unfed for 5 weeks. In that study, the authors concluded that metabolic reserves play a role in activity levels, and insects running low of these reserves might employ energy-conserving strategies such as reducing movements. Further, in an experiment using a related bed bug species, *Cimex hemipterous*, researchers found that blood-fed females showed significantly greater movement distances than unfed females [35]. This increased activity of recently-fed bed bugs might be to take a risk and seek new harborages or oviposition sites since they have plenty of energy reserves.

### 3.3. Diffusion Rates

Mean diffusion rates estimated using initial condition (IC_1_) are listed in Table 2. A two-tail *t*-test of these mean diffusion rates showed no statistical significance between one-dimensional and two-dimensional arenas (pooling all fed and unfed mean diffusion rates by their arena dimension). These mean diffusion rates also failed to show statistical significance between fed and unfed status over each arena dimension (pooling all one- and two-dimensional arena mean diffusion rates by their status). As indicated by the magnitude of the standard deviations observed in each rep, there was considerable variability in the estimated diffusion rates in all reps.

**Table 2:** Mean diffusion rates using (IC_1_) in cm^2^/s of fed *vs.* unfed bed bugs in one- and two-dimensional arenas, standard deviation in parentheses.

Status	Rep 1	Rep 2	Rep 3	Rep 4	Rep 5	Overall
one-dimensional fed	0.040 (0.008)	0.006 (0.004)	0.071 (0.076)	0.079 (0.050)	0.138 (0.140)	0.070 (0.051)
one dimensional unfed	0.014 (0.008)	0.022 (0.011)	0.060 (0.079)	0.009 (0.005)	0.013 (0.008)	0.024 (0.021)
two-dimensional fed	0.143 (0.146)	0.006 (0.007)	0.006 (0.012)	0.055 (0.063)	0.059 (0.076)	0.054 (0.056)
two-dimensional unfed	0.051 (0.068)	0.017 (0.024)	0.004 (0.011)	0.062 (0.055)	0.023 (0.019)	0.032 (0.024)

**Figure 7:**
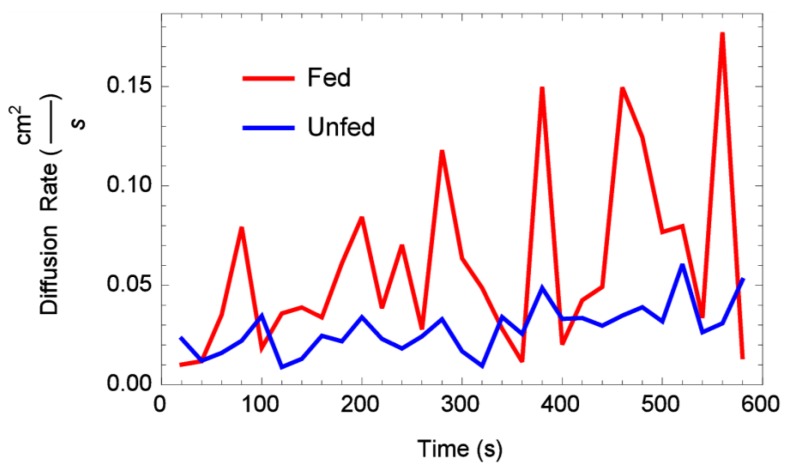
Estimated mean diffusion rate through time, fed *vs.* unfed bed bugs, one-dimensional arena.

**Figure 8:**
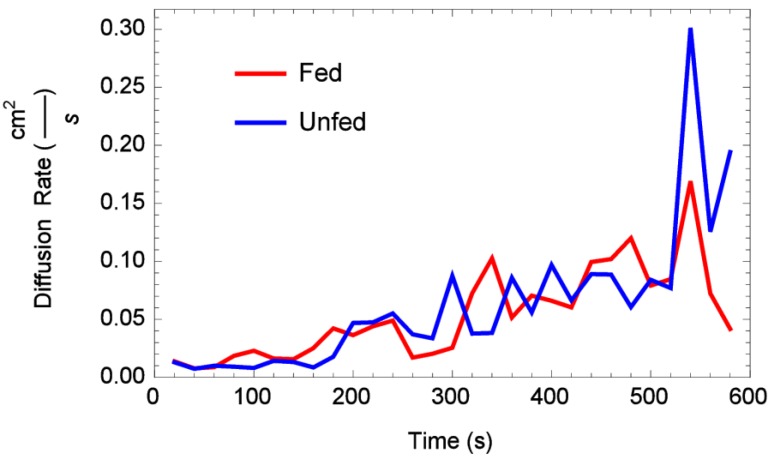
Estimated mean diffusion rate through time, fed *vs.* unfed bed bugs, two-dimensional arena.

Time-dependent diffusion rates obtained using initial condition (IC_2_) are plotted *versus* time in Figure 7 and Figure 8 for one- and two-dimensional arenas, respectively. Note that both figures display mean diffusion rates taken over all reps by status. Results of a repeated measures ANOVA performed on the one-dimensional arena diffusion rates showed that the factors status, time, and status-time interactions were all shown not to be statistically significant (*F*-value 1.55, *p* < 0.248). Results of a repeated measures ANOVA performed on the two-dimensional arena diffusion rates showed that status and status-time interactions were also not statistically significant (*F*-value 0.054, *p* < 0.823). However, time was shown to be statistically significant *p* < 0.000806. In fact, Figure 8 shows that in the two-dimensional arena both fed and unfed diffusion rates were almost identical, but showed an increase in the diffusion rate over time.

**Figure 9:**
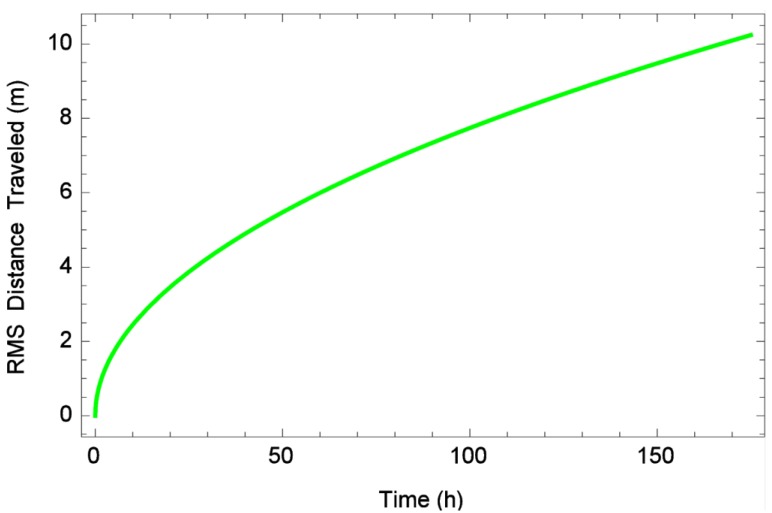
Time (h) *versus* predicted root mean squared distance (m) traversed by an individual bed bug dispersing at the maximum (over all reps) observed diffusion rate of 0.416 cm^2^/s.

Overall, the estimated diffusion rate of bed bugs in arenas in this study ranged from 0.00006 cm^2^/s to 0.416 cm^2^/s (see Table 2 for means), which is almost no movement during the 10 min observation period to a predicted root mean squared distance of approximately 19 centimeters per 10 min (see Figure 9). This result is somewhat similar to another study [22] which found that bed bugs in the absence of host cues moved approximately 10 cm in 10 min. It is important to note that bed bugs are often not moving. Diffusion rates provided here include times of non-movement during our observation periods. At the maximum diffusion rate observed in this study, and without presence of human scents or attractants, to achieve a root mean squared distance of 10 meters would take bed bugs over 166 h. See Figure 9 for a graph of time *versus* predicted root mean squared distance traversed by an individual bed bug dispersing at the maximum diffusion rate observed in this study. Certainly, in the presence of human cues or perhaps even in the dark, bed bugs might take a more directional path and thus shorten this time frame.

## 4. Conclusions

Herein we report dispersal patterns for bed bugs, both fed and unfed, in two different arena types during 10-min observation periods. We also report for the first time mathematical diffusion rates for bed bugs in these arenas. Interestingly, these diffusion rate calculations suggest that, upon initial introduction into a new area, bed bugs would have a difficult time traversing long distances when left alone to “randomly” disperse. What happens over longer periods of time, such as days or weeks, remains unknown. The presence of human scents and/or other environmental conditions may alter those rates. These data also support the idea that the rate of bed bug spread in a dwelling is not attributable to active dispersal alone. Further investigation is needed to elucidate bed bug dispersal patterns and the various factors which influence those behaviors.
